# Development of a Prediction Model for Colorectal Cancer among Patients with Type 2 Diabetes Mellitus Using a Deep Neural Network

**DOI:** 10.3390/jcm7090277

**Published:** 2018-09-12

**Authors:** Meng-Hsuen Hsieh, Li-Min Sun, Cheng-Li Lin, Meng-Ju Hsieh, Kyle Sun, Chung-Y. Hsu, An-Kuo Chou, Chia-Hung Kao

**Affiliations:** 1 Department of Electrical Engineering and Computer Sciences, University of California, Berkeley, CA 94720, USA; emersonhsieh@berkeley.edu; 2 Department of Radiation Oncology, Zuoying Branch of Kaohsiung Armed Forces General Hospital, Kaohsiung 81342, Taiwan; limin.sun@yahoo.com; 3 Management Office for Health Data, China Medical University Hospital, Taichung 40447, Taiwan; orangechengli@gmail.com; 4 College of Medicine, China Medical University, Taichung 40402, Taiwan; edmundchou@gmail.com; 5 Department of Medicine, Poznan University of Medical Sciences, 61-701 Poznań, Poland; 76519@student.ump.edu.pl; 6 Program of Computer Science, Arizona State University, Tempe, AZ 85287, USA; yaomon18@yahoo.com; 7 Graduate Institute of Biomedical Sciences, China Medical University, Taichung 40402, Taiwan; hsucy63141@gmail.com; 8 Department of Anesthesiology, China Medical University Hospital, Taichung 40447, Taiwan; 9 Department of Nuclear Medicine and PET Center, China Medical University Hospital, Taichung 40447, Taiwan; 10 Department of Bioinformatics and Medical Engineering, Asia University, Taichung 41354, Taiwan

**Keywords:** type 2 diabetes mellitus, colorectal cancer, deep neural network, the national health insurance database, receiver operating characteristic

## Abstract

Objectives: Observational studies suggested that patients with type 2 diabetes mellitus (T2DM) presented a higher risk of developing colorectal cancer (CRC). The current study aims to create a deep neural network (DNN) to predict the onset of CRC for patients with T2DM. Methods: We employed the national health insurance database of Taiwan to create predictive models for detecting an increased risk of subsequent CRC development in T2DM patients in Taiwan. We identified a total of 1,349,640 patients between 2000 and 2012 with newly diagnosed T2DM. All the available possible risk factors for CRC were also included in the analyses. The data were split into training and test sets with 97.5% of the patients in the training set and 2.5% of the patients in the test set. The deep neural network (DNN) model was optimized using Adam with Nesterov’s accelerated gradient descent. The recall, precision, F_1_ values, and the area under the receiver operating characteristic (ROC) curve were used to evaluate predictor performance. Results: The F_1_, precision, and recall values of the DNN model across all data were 0.931, 0.982, and 0.889, respectively. The area under the ROC curve of the DNN model across all data was 0.738, compared to the ideal value of 1. The metrics indicate that the DNN model appropriately predicted CRC. In contrast, a single variable predictor using adapted the Diabetes Complication Severity Index showed poorer performance compared to the DNN model. Conclusions: Our results indicated that the DNN model is an appropriate tool to predict CRC risk in patients with T2DM in Taiwan.

## 1. Introduction

Diabetes mellitus (DM) is a common chronic disease worldwide. According to the Global Report on Diabetes from the World Health Organization, the prevalence of DM has been steadily rising for the past three decades, becoming a major public health issue. In 2014, 422 million people in the world had diabetes—8.5% of the adult population [1]. In Taiwan, the standardized incidence rate of DM is in accordance with the global trend, with a near constancy (1.043% in 2000 and 1.160% in 2009 among age 20–79 residents). However, its prevalence has steadily increased (4.31% in 2000 and 6.38% in 2009 among age 20–79 residents), suggesting a possibility of better DM care that leads to a decrease in mortality rates of patients with DM [2].

As the number of patients with chronic DM increases, certain diseases related to DM become concerns among these patients. Studies have suggested that patients with DM are at a higher risk to develop cancer overall and several individual cancers compared to the general population [3,4,5,6,7,8,9]. The risk of developing colorectal cancer (TaCRC) among patients with DM was revealed by earlier reports [10,11,12,13].

Cancer has been Taiwan’s leading cause of mortality since 1982 and CRC has been the most common type of malignancy recorded in the country since 2006 [14]. In 2015, the age-adjusted incidence rate of CRC in Taiwan was 43.58/100,000 people, an increase from 2005 of 20.9% and 8.3% for men and women, respectively [15]. CRC has also been ranked as the third leading cause of cancer-related death in Taiwan, from 2013 to 2017, for both men and women, as well as both combined. Consequently, cancer continues to be a challenge for the public health field of Taiwan. It has come to our government’s attention, resulting in population-based investigations regarding early diagnosis and cancer-preventive epidemiology. Based on this concern and the suggestion of a possible link between CRC risk and type 2 DM (T2DM) by previous researchers, we proposed this study aimed at creating a deep neural network (DNN) to predict the onset of CRC among patients with T2DM in Taiwan.

## 2. Experimental Section

### 2.1. Data Source and Sampled Participants

This study was approved by the Research Ethics Committee of the China Medical University and Hospital in Taiwan (CMUH104-REC2-115-CR3). For the present study, we used a subset of data from the National Health Insurance Research Database (NHIRD) and the Longitudinal Cohort of Diabetes Patients (LHDB), which contains health data of 1,700,000 patients with newly diagnosed T2DM (International Classification of Diseases, Ninth Revision, Clinical Modification (ICD-9-CM) code 250.x0 and 250.x2) from 2000–2012. Subjects that had at least two diagnoses of T2DM within a year were eligible for inclusion in the LHDB. The first diagnosis date was defined as the index date of T2DM. T2DM patients with a CRC history (ICD-9-CM 153, 154) before the index date, aged less than 20 years and with incomplete information on demographics were excluded.

### 2.2. Outcome Measurements, Comorbidities, and Medications

All study subjects were followed from the index date to the date of CRC diagnoses, date of withdrawal from the insurance program, or the end of 2013; whichever came first. The baseline comorbidities considered in this study included hypertension, hyperlipidemia, stroke, congestive heart failure, colorectal polyps, obesity, chronic obstructive pulmonary disease (COPD), coronary artery disease (CAD), asthma, smoking (stop-smoking clinic), inflammatory bowel disease, irritable bowel syndrome, alcohol-related illness, and chronic kidney disease. The adapted Diabetes Complication Severity Index (aDCSI) score consists of scores from 7 complication categories including retinopathy, nephropathy, neuropathy, cerebrovascular, cardiovascular, peripheral vascular disease and metabolic; it ranges from 0 to 13 [16,17]. Medications that may be associated with CRC were also evaluated, including statin, insulin, sulfonylureas, metformin, thiazolidinedione (TZD), and other antidiabetic drugs.

### 2.3. Constructing Training and Data Sets

The data comprised of 1,349,640 data points, each representing one patient. The data had 37 input features and 2 output features. The positive output class represented the diagnosis of CRC, while the negative output class represented no diagnosis. The data were split into training and test sets: 97.5% of the data were used as the training set and 2.5% were used as the test set. This ratio was chosen both to have a sufficient number of data points for validation and to use the majority of the dataset for training. The data points were randomly allocated to each set. Table 1 shows the allocation between the training and test sets.

### 2.4. Algorithm and Training

The average *k*-fold cross-validation accuracy, with a *k*-value of 10, was used as the metric to determine the best hyperparameters, optimizers, and loss functions of predictors.

The DNN model is a multilayer perceptron deep neural network. The model used here consisted of one input layer of 37 dimensions, three hidden layers of 30 dimensions, and a scalar output layer. The number of dimensions corresponds to the number of artificial neurons in each layer. Each layer was densely connected, meaning that the neurons of each layer were connected to neurons of the preceding and successive layers. The model was trained using a stochastic gradient descent, an iterative algorithm used to optimize the weights of neurons in the network, with a mini-batch size of 128. The model was optimized using Adam with Nesterov’s accelerated gradient descent [18,19,20]. The input and hidden layers used a Rectified Linear Unit (ReLU) activation function [21], while the output layer used the Softmax activation function. These activation functions were applied to the output of each neuron. A dropout, a regularization technique used to prevent overfitting, of 20% was applied to the input layer and each hidden layer [22]. The categorical cross entropy function was used as the loss function. The neuron weights were initialized using normalized He initialization [23].

A non-diagnosis of CRC was prevalent in the data set. Each data point in the positive class was weighted approximately 40 times greater than each data point in the negative class to ensure that the output of the prediction was not unbalanced towards the dominant class.

The software was implemented using Python (version 3.7.0) [24]. The DNN model was created and trained with the Tensorflow framework (version 1.9.0) [25].

### 2.5. Statistical Analyses

Distributions of socio-demographic factors, including age, gender, urbanization level, occupation, underlying disease, diabetes complication, and medications of the patient with CRC and without CRC were compared. A Chi-square test and a *t*-test were used to test the difference between categorical and continuous variables, respectively, between the two groups.

Accuracy was not a reliable measurement of predictor performance due to the unbalanced data distribution [26]. Instead, we used the weighted average recall (sensitivity), precision (positive predictive value), and F_1_ (harmonic mean of sensitivity and precision) values to evaluate predictor performance. These three metrics all have ideal values of one. The F_1_, precision, and recall values were calculated for the training set, test set, and all data using the scikit-learn library.

Additionally, the receiver operating characteristic (ROC) curve was used as a metric to measure predictor performance. The ROC was calculated between the outcome and the predicted probability of the outcome by the DNN model. The ROC curve was also computed using aDCSI as the sole predictor. The area under the ROC curve (AUROC) for the DNN model and aDCSI were compared to determine the performance of the DNN model and both values were also compared to the ideal value of 1 and to the null hypothesis area of 0.5 [27]. The ROC curve was calculated using IBM SPSS. Data management was performed using the SAS 9.4 software (SAS Institute, Cary, NC, USA). All *P*-values were 2 tailed and a *p*-value < 0.05 was considered significant.

## 3. Results

### 3.1. Demographic Features of Patients

Eligible study participants consisted of 1,349,640 T2DM patients, 14,867 of whom were with CRC and 1,334,773 of whom were without CRC (Table 2). The mean age was 63.7 years (SD = 11.2 years) for the CRC group and was 56.2 years (SD = 14.2 years) for the non-CRC group. There were more men than women. The two groups preferred to reside in urbanized areas (58.9% vs. 58.8%). Most of the occupations in both groups were white-collar jobs (45.0% vs. 48.1%). The comorbidities of hypertension, congestive heart failure, colorectal polyps, COPD, CAD, and irritable bowel syndrome were significantly higher in the CRC group than in the non-CRC group. The T2DM-related cardiovascular complication was more prevalent in the CRC group than in the non-CRC group. The mean aDCSI score at the end of the follow-up was 2.75 (SD = 2.15) in the CRC group and 3.03 (SD = 2.35) in the non-CRC group. All medications listed in Table 2 had higher proportions in the non-CRC group than in the CRC group. The mean follow-up periods were 4.73 (SD = 3.33) years in the CRC group and 6.86 (SD = 3.87) years in the non-CRC group.

### 3.2. Evaluation of Predictor Performance

**Figure 1:**
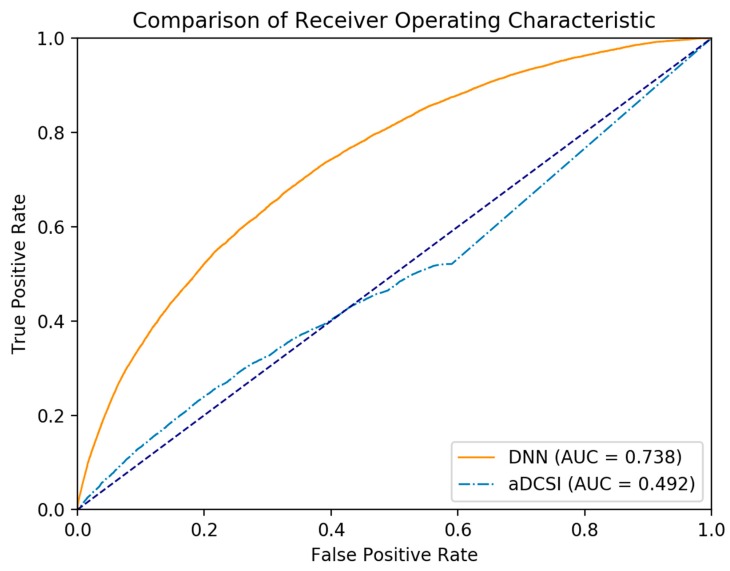
The ROC curve of the DNN model and aDCSI model in predicting CRC.

The F_1_, precision, the recall values and the AUROC of the DNN model across all data are outlined in Table 3. The AUROC of aDCSI is outlined in Table 4. Figure 1 shows the ROC curve of the DNN model and aDCSI model for predicting CRC.

The AUROC of the aDCSI across all three datasets was close to the null hypothesis area of 0.5, which showed that aDCSI alone cannot be used as a predictor for CRC. This signified a necessity for a multivariate prediction model that takes into account all variables. The AUROC of the DNN model across all three datasets was significantly greater than the null hypothesis area and the AUROC of the aDCSI.

The DNN model had a high precision value across the test set (0.980), which indicated that the DNN model had a relatively low false positive rate. While the recall value across the test set was lower than the precision value, the recall value was also relatively high (0.886), which signified a low false negative rate.

## 4. Discussion

This national population-based study demonstrated that the DNN model appropriately predicted CRC. In contrast, a single variable predictor using aDCSI showed poorer performance compared to the DNN model.

Earlier studies suggested that compared to the general population, patients with DM are at a higher risk to develop CRC [10,11,12,13]. Although DM and cancer share several risk factors, such as obesity, aging, unhealthy food and physical inactivity [6], the association between DM and the risk of CRC is biologically plausible based on the findings of previous studies. The potential mechanisms contributing to the development of diabetes-associated CRC may include insulin resistance and associated hyperglycemia, hyperinsulinemia, oxidative stress, and chronic inflammation [6,11,28]. Insulin stimulates cell proliferation and most cancer cells express the insulin-like growth factor (IGF) receptor [28,29]. The IGF system is a potent growth regulator closely linked with carcinogenesis [30] and several observational studies and reviews have revealed a linkage between elevated IGF levels and the risks of adenomatous polyps or CRC [31,32,33,34].

In Taiwan, by using the NHIRD, several authors used traditionally statistical methods to assess the cancer risk among patients with T2DM and anti-diabetic therapies [9,35,36,37]. Hsieh applied logistic regression models to test the risk of T2DM and antidiabetic drugs on cancers. They found that there was a significantly higher risk for CRC (adjusted odds ratio = 1.206, 95% confidence interval = 1.142–1.274) in patients with T2DM [9]. Chiu employed a Cox proportional hazards regression analysis to evaluate T2DM and antidiabetic drugs with the risk of gastrointestinal malignancy. They indicated that T2DM was significantly associated with an increased risk of CRC (adjusted hazard ratio: 1.58, 95% confidence interval = 1.28–1.94) [35]. Tseng created multivariable Cox regression models to calculate the adjusted relative risk of T2DM on CRC and concluded that a significantly higher risk was detected [36]. Our team carried out Cox regression analyses several years ago to determine if TZD can decrease cancer risk in T2DM patients and highlighted that no significant difference was observed for the risk of CRC in the TZD group relative to the non-TZD group [37]. To further clarify this issue, the current study attempted to use DNN to develop a prediction model for CRC among patients with T2DM. In addition, the drug effects were also considered for the analyses. A DNN is an artificial neural network with multilayer perceptron and uses sophisticated mathematical modelling to process data in complex ways [38]. It can be used for prediction, forecasting, diagnosis, and decision making in different fields including the healthcare field.

We found that the AUROC of the DNN was significantly greater than that of using only aDCSI. We used aDCSI as a single variable predictor and the AUROC of aDCSI in predicting CRC was close to the null hypothesis area of 0.5, showing that using aDCSI to predict CRC was not effective. In contrast, DNN was designed for a multivariate predictor and the AUROC of the DNN model was significantly greater than 0.5, indicating that a DNN model can be an effective prediction model for CRC. Our government has already launched a nationwide screening program of CRC since 2004 and a free fecal immunochemical test is offered biennially to individuals aged 50 to 75 [39]. According to our findings, the program may extend to cover patients with T2DM beyond this age range.

Table 2 indicates that T2DM patients with hypertension, congestive heart failure, colorectal polyps, COPD, CAD, and irritable bowel syndrome had a significantly higher risk of CRC compared with T2DM patients without the corresponding underlying diseases. On the contrary, T2DM patients with hyperlipidemia, obesity, smoking, alcohol-related illness and CKD had a significantly lower risk of CRC compared with T2DM patients without the corresponding underlying diseases. As all the listed comorbidities were suggested risk factors for either T2DM or CRC or both, the findings presented here may represent the effects of intricate mechanisms among risk factors, T2DM and CRC.

The main strength of this study lies in a use of a population-based cohort with a large and nationally representative sample, which increases its generalizability in Taiwan. However, we have to acknowledge several limitations as below. Firstly, detection bias could exist because patients with T2DM are supposed to have more clinical visits than the general population and Lewis found that diabetic patients receiving medications, particularly in the first year of diagnosis, are more likely to undergo lower endoscopic examinations [40] and we can expect an overestimation of the incidence of CRC among these groups. However, Taylor stated that diabetic patients have a significantly poorer response to colonoscopy bowel cleansing preparations than nondiabetic patients [41] and it might lead to the decreased detection of CRC, resulting in an underestimation of the relationship between T2DM and CRC [11]. Secondly, inherent limitations of NHIRD hinder our ability to get some information related to the T2DM and CRC, such as smoking habits, alcohol consumption, body mass index (BMI), family history of T2DM or CRC, diet, and physical activity. To deal with this, we tried to use the comorbidities as surrogates for some risk factors of CRC, such as COPD and a stop-smoking clinic for smoking, alcohol-related illness for alcohol, and obesity for BMI. However, we should admit that the use of these comorbidities as surrogate risk factors for CRC did not allow any interpretation of data. Finally, unlike the traditional Cox proportional hazard model, our predictive models could not provide valued levels (e.g., 95% confidence intervals and *p*-values) to evaluate statistical significance; instead, we used recall, precision, F_1_, and AUROC to determine the predictor performance. The AUROC is 0.74 for the DNN, which is acceptable. Although these limitations suggest cautious interpretation of the results of the study, the diagnosis of T2DM and CRC is highly reliable which makes our results more convincing.

## 5. Conclusions

In conclusion, based on the DNN predictive model, our findings suggest that Taiwanese patients with T2DM were at an increased risk of developing CRC. Although we have a relatively successful screening policy for CRC, our findings might encourage the government to consider a slight policy modification regarding screening guidelines to include patients with T2DM with a wider age range.

## Figures and Tables

**Table 1:** Distribution of training and test sets.

	All Patients	Training Set	Test Set
*N*	1,349,640	1,315,899	337,410

**Table 2:** Baseline characteristics of T2DM patients with and without colorectal cancer.

	Colorectal Cancer				
	No		Yes		
	*N* = 1334773		*N* = 14867		
Variable	*n*	(%)	*n*	(%)	*p* Value
Age group (year)					<0.001
≤49	420,354	31.5	1737	11.7	
50–64	515,804	38.6	5950	40.0	
65 +	398,615	29.9	7180	48.3	
Mean (SD) (year) †	56.2	14.2	63.7	11.2	<0.001
Gender					<0.001
Women	633,366	47.5	6259	42.1	
Men	701,407	52.6	8608	57.9	
Urbanization level #					0.001
1 (highest)	387,470	29.0	4374	29.4	
2	397,750	29.8	4383	29.5	
3	223,753	16.8	2337	15.7	
4 (lowest)	325,800	24.4	3773	25.4	
Occupation					<0.001
White collar	640,808	48.1	6695	45.0	
Blue collar	554,764	41.6	6577	44.2	
Others ‡	139,201	10.4	1595	10.7	
Underlying disease					
Hypertension	984,221	73.7	11,707	78.7	0.001
Hyperlipidemia	899,397	67.4	9102	61.2	<0.001
Stroke	259,808	19.5	2940	19.8	0.34
Congestive heart failure	183,790	13.8	2076	14.0	<0.001
Colorectal polyps	58,952	4.42	1562	10.5	<0.001
Obesity	71,119	5.33	452	3.04	<0.001
COPD	375,331	28.1	4654	31.3	<0.001
CAD	510,862	38.3	6264	42.1	<0.001
Asthma	259,565	19.5	2859	19.2	0.51
Smoking	50,660	3.80	324	2.18	<0.001
Inflammatory bowel disease	49,295	3.69	575	3.87	0.26
Irritable bowel syndrome	182,951	13.7	2781	18.7	<0.001
Alcohol-related illness	142,265	10.7	1107	7.45	<0.001
CKD	856,446	64.2	8314	55.9	<0.001
Diabetes complication (components of the aDCSI)					
Retinopathy	262,293	19.7	2423	16.3	<0.001
Nephropathy	479,819	36.0	4659	31.3	<0.001
Neuropathy	398,979	29.9	3871	26.0	<0.001
Cerebrovascular	354,430	26.6	3741	25.2	<0.001
Cardiovascular	769,763	57.7	8887	59.8	<0.001
Peripheral vascular disease	365,797	27.4	3406	22.9	<0.001
Metabolic	60,532	4.54	434	2.92	<0.001
Mean aDCSI score (SD) †					
Onset	1.55	1.67	1.55	1.62	0.74
End of follow-up	3.03	2.35	2.75	2.15	<0.001
Medications					
Statin	706,079	52.9	6351	42.7	<0.001
Insulin	437,994	32.8	3506	23.6	<0.001
Sulfonylureas	770,838	57.8	8432	56.7	<0.001
Metformin	856,446	64.2	8314	55.9	<0.001
TZD	223,650	16.8	1767	11.9	<0.001
Other antidiabetic drugs	365,662	27.4	3071	20.7	<0.001
Mean follow-up for endpoint, y (SD) †	6.86	3.87	4.73	3.33	<0.001

#: The urbanization level was categorized by the population density of the residential area into 4 levels, with level 1 as the most urbanized and level 4 as the least urbanized. ‡: Other occupations included primarily retired, unemployed, or low income populations. aDCSI = adapted Diabetes Complication Severity Index. Chi-square test, and †: *t*-test comparing subjects with and without death.

**Table 3:** Performance of DNN across all data, the training set, and the test set.

Dataset	F_1_	Precision	Recall	AUROC	AUROC 95% CI	AUROC SE
All data (*n* = 1349640)	0.931	0.982	0.889	0.738	0.734–0.742	0.002
Training set (*n* = 1315899)	0.931	0.982	0.889	0.739	0.735–0.743	0.002
Test set (*n* = 337410)	0.929	0.980	0.886	0.700	0.674–0.727	0.013

**Table 4:** The receiver operating characteristic of aDCSI.

Dataset	AUROC	AUROC 95% CI	AUROC SE
All data (*n* = 1349640)	0.492	0.487–0.497	0.003
Training set (*n* = 1315899)	0.492	0.487–0.498	0.003
Test set (*n* = 337410)	0.498	0.466–0.530	0.016

## Abbreviations

T2DM: type 2 diabetes; NHIRD: National Health Insurance Registration Database; ICD-9-CM: International Classification of Diseases, Ninth Revision, Clinical Modification; DNN: deep neural network; SD: standard deviation. Cancer Registry. Available online: http://tcr.cph.ntu.edu.tw/main.php?Page=A5B2 (accessed on 25 July 2018).
